# Precipitation Processes in Sanicro 25 Steel at 700–900 °C: Experimental Study and Digital Twin Simulation

**DOI:** 10.3390/ma18153594

**Published:** 2025-07-31

**Authors:** Grzegorz Cempura, Adam Kruk

**Affiliations:** Faculty of Metals Engineering and Industrial Computer Science, Centre of Electron Microscopy for Materials Science, AGH University of Krakow, al. Mickiewicza 30, 30-059 Krakow, Poland; kruczek@agh.edu.pl

**Keywords:** CALPHAD, heat treatment, SEM, TEM, microstructure, austenitic steels, high temperature materials

## Abstract

Sanicro 25 (X7NiCrWCuCoNb25-23-3-3-2) steel is specifically designed for use in superheater components within the latest generation of conventional power plants. These power plants operate under conditions often referred to as super-ultra-supercritical, with steam parameters that can reach up to 30 MPa and temperatures of 653 °C for fresh steam and 672 °C for reheated steam. While last-generation supercritical power plants still rely on fossil fuels, they represent a significant step forward in more sustainable energy production. The most sophisticated facilities of this kind can achieve thermodynamic efficiencies exceeding 47%. This study aimed to conduct a detailed analysis of the initial precipitation processes occurring in Sanicro 25 steel within the temperature range of 700–900 °C. The temperature of 700 °C corresponds to the operational conditions of this material, particularly in secondary steam superheaters in thermal power plants that operate under ultra-supercritical parameters. Understanding precipitation processes is crucial for optimizing mechanical performance, particularly in terms of long-term strength and creep resistance. To accurately assess the microstructural changes that occur during the early stages of service, a digital twin approach was employed, which included CALPHAD simulations and experimental heat treatments. Experimental annealing tests were conducted in air within the temperature range of 700–900 °C. Precipitation behavior was simulated using the Thermo-Calc 2025a with Dictra software package. The results from Prisma simulations correlated well with the experimental data related to the kinetics of phase transformations; however, it was noted that the predicted sizes of the precipitates were generally smaller than those observed in experiments. Additionally, computational limitations were encountered during some simulations due to the complexity arising from the numerous alloying elements present in Sanicro 25 steel. The microstructural evolution was investigated using various methods, including light microscopy (LM), scanning electron microscopy (SEM), and transmission electron microscopy (TEM).

## 1. Introduction

The policy of most countries is to reduce fuel consumption, especially of fossil fuels. Reducing the consumption of fossil fuels while maintaining adequate generating capacity can be achieved through several methods, including energy conservation, the use of nuclear power plants for power generation, and the utilization of biomass [1,2]. Currently, the bulk of electricity is generated using conventional power plants. The complete replacement of conventional power plants, especially as global energy consumption continues to rise, may be very difficult to implement in practice [3]. One way to reduce consumption may be the use of high-efficiency conventional power plants operating at supercritical and ultra-supercritical parameters. The use of power plants of this type can enable the construction of power plants with proven technology, high availability, and higher efficiency than most power plants currently in operation. This may make it possible, in the interim period, to generate energy at low or moderate prices for both the construction of the power plant itself and for the competitive price of the energy produced, while reducing CO_2_ emissions [1,2,3,4,5,6,7]. The construction of high-efficiency power plants requires the use of heat-resistant materials, such as modern austenitic steels. Sanicro 25 austenitic steel (X7NiCrWCuCoNb25-23-3-3-2) is designed for making steam superheater components in conventional power plant systems. The operating temperature of this material is about 700 °C. Most papers published on Sanicro 25 deal with samples that have been heat-treated for extended periods, characterizing the microstructure of Sanicro 25 after long-term aging [8,9,10,11,12,13]. There are also quite a large number of papers dealing with the oxidation of this steel [12,14,15,16,17,18] and the influence of the welding [19,20] on its properties.

Within this work, we focus on precipitation processes that occur in the first hours after standard heat treatment of the material. We also discuss the impact of heat treatment on the as-received state. To achieve our goals, we utilize Thermocalc simulations, experimental heat treatment, and detailed characterization of the materials’ microstructure at various stages, including both the as-received state and after high-temperature annealing. The purpose of the research presented in this paper is to conduct a detailed analysis of the initial precipitation processes occurring in Sanicro 25 steel within the temperature range of 700 °C to 900 °C. The temperature of 700 °C corresponds to the application temperature of this material, for example, in secondary steam superheaters in thermal power plants operating at supercritical parameters. Precipitation processes are crucial for strength, particularly in terms of long-term stability and creep resistance [21,22]. To accurately determine the microstructural changes occurring during the initial period of operation, the precipitation processes were simulated using Dictra-Thermocalc packages. Additionally, experimental annealing studies were conducted on the samples within a temperature range of 700 °C to 900 °C, and microstructural analyses were performed using light microscopy, scanning electron microscopy, and transmission electron microscopy techniques. Microstructural studies enable the detection of defects that may be present in the material, such as pores, inclusions, and the presence of phases other than the desired ones. Material testing after tests or actual long-term operation enables the optimization of heat treatment processes or, if necessary, the optimization of the material’s operating conditions. Such tests also allow the extension of the service life of high-temperature materials in cases where it is required to validate the microstructure and determine whether the material can continue to be used without compromising the safety of the equipment or installation being operated.

## 2. Materials and Methods

### 2.1. Material

Sanicro 25 steel (X7NiCrWCuCoNb25-23-3-3-2) was used in this study. The chemical composition of Sanicro yields a steel matrix comprising a solid solution, whose strength is derived from solid-solution strengthening effects and the presence of precipitates formed during both heat treatment and service conditions. Its chemical composition is shown in Table 1.

### 2.2. Thermodynamic Simulations

Within the framework of the present work, thermodynamic simulations of precipitation processes and phase changes during annealing of Sanicro 25 steel were carried out using the Thermo-Calc program, version 2025a [23]. This program is used to determine thermodynamic equilibrium in multicomponent systems. Thermo-calc employs numerical methods to determine the minimum of the global free energy, which corresponds to thermodynamic equilibrium. This allows it to predict both the stable phases at the analyzed temperature and their corresponding chemical composition [24,25]. Dictra (Diffusion-Controlled Transformation) is a specialized add-on to Thermo-Calc that enables the simulation of diffusion processes in multicomponent materials. Calculations include thermodynamic processes, such as the stability of phases at the analyzed temperature and the kinetics of phase transformations resulting from diffusion processes. Dictra performs calculations based on the diffusion equations of multicomponent systems and solves them iteratively in small time steps. For this reason, the calculations are computationally intensive, and their complexity increases with the number of elements and the number of phases present in a given system. The Scheil calculation method (also known as the Scheil–Gulliver model) was used to determine the chemical and phase composition of Sanicro 25 after the solidification process. This method is used to simulate the solidification process of metallic alloys under highly segregated conditions. The approach assumes that as the alloy crystallizes, successive portions of the solid phase are formed in equilibrium with the liquid phase. Still, the already formed solid phase does not undergo further diffusive exchange of components. This means that in this type of calculation, the chemical composition of once-crystallized crystals is “frozen” and cannot diffusively equilibrate with subsequent portions of the solid phase. In real conditions during crystallization, diffusion processes can occur. Compared to equilibrium solidification (yielding a homogeneous composition and a narrower solidification range), Scheil’s calculations indicate a wider solidification range and strong microsegregation of dopants than do the calculations by Stojan et al. [26]. For Sanicro 25 steel, these calculations suggest a range of existence of the liquid phase that is lower by more than 80 °C (the liquid phase disappears only around 1240 °C), compared to cooling under equilibrium conditions [12]. Simulation studies performed in the present work aimed to determine the effect of annealing for one hour at temperatures of 750, 800, 850, and 900 °C on the chemical composition of the matrix and its phase composition after this process.

### 2.3. Experiment—Heat Treatment

The test specimens were cut from a pipe made of Sanicro 25 steel after standard heat treatment. The specimens had dimensions of about 9 × 9 × 2 mm. The experimental annealing process was conducted in an air atmosphere, and the sample was immediately cooled in water after the process.

Despite the ever-improving techniques that enable the numerical simulation of a heat-treated material’s microstructure, testing samples after actual forming or heat-treating processes remains necessary.

### 2.4. Microstructural Characterization of Samples After Heat Treatment

To verify the validity of the thermodynamic simulation results with the exact phase composition of Sanicro 25, microstructural studies of the samples in their as-delivered state and after heat treatment were conducted using optical microscopy (LM), scanning electron microscopy (SEM), and transmission electron microscopy (TEM) techniques.

Samples for optical microscopy examination were cut using a circular diamond saw, with continuous water cooling during the cutting process. After cutting, the samples were embedded in an electrically conductive thermoplastic resin. Conductive resin was used because the same specimens were subsequently analyzed using both optical microscopy and scanning electron microscopy. After embedding, the metallographic cross-sections were ground using abrasive papers, starting from a grit size of 400 and progressing up to 2000. Following grinding, the samples were polished using an Al_2_O_3_ suspension.

The prepared specimens were examined with a Zeiss Axio Imager M1m optical microscope (Oberkochen, Germany). The use of light microscopy allowed for an overview of the samples. The samples prepared as described above were also examined using a high-resolution Schottky Field Emission Gun (FEG) scanning electron microscope, Merlin Gemini 2 of Zeiss (Oberkochen, Germany), equipped with a Quantax 800 energy-dispersive X-ray spectrometer by Bruker (Berlin, Germany). The Merlin Gemini 2 scanning electron microscope is equipped with several detection systems, including an on-axis in-lens secondary electron (SE) detector.

Chemical microanalysis and elemental maps were performed using the SEM-EDS technique on unetched samples to avoid the influence of the etchant on the chemical microanalysis results. Since the chemical composition of Sanicro 25 also contains heavy elements, a 20 kV high voltage was applied to ionize these elements as well. To determine the volume of interaction of the electron beam with the sample, CASINO v3 software was used [27]. After collecting SEM-EDS elemental maps, to enhance the contrast, the samples were electrochemically etched in a 10% oxalic acid solution using a voltage of approximately 6 V for 20–30 s. After this process, the samples were once again observed using LM and SEM techniques. The ImageJ Version 1.54 [28] software package was used to characterize the microstructure observed with LM and SEM.

To determine microstructural features at higher resolution, the TEM technique was employed. TEM studies were carried out on FIB-SEM lamellae prepared with a Zeiss Crossbeam 350, (Oberkochen, Germany) and also on thin films prepared by electropolishing using a Tenupol-5 device from Struers (Ballerup, Denmark). The following conditions were used: electrolyte A2 from Struers, a temperature of 10 °C, and a voltage of 25 V. The process of electropolishing was automatically stopped when a photodiode detected a hole in the material. During TEM studies, it was found that specimens prepared using the FIB-SEM technique provided higher-quality results and allowed for more detailed analyses. Therefore, mainly this type of sample was used for TEM investigations. A Titan G2 transmission electron microscope from FEI (Eindhoven, The Netherlands), equipped with a spherical aberration corrector for the beam-forming system and a ChemiSTEM characteristic X-ray spectrometer, was used for the study. TEM investigations were performed using bright field (BF) TEM, Selected Area Electron Diffraction (SAED), Bright Field Scanning Transmission Electron Microscopy (BF-STEM), and High Angle Annular Dark Field Scanning Transmission Electron Microscopy (HAADF-STEM). Digital Micrograph 3.6 [29] and TIA 4.17 [30] software packages were used to analyze the TEM images. Analysis of the collected SAED patterns was completed using JEMS V2 [31] simulation software. Quantitative results of chemical composition were obtained with the Esprit 1.9 package from Brucker (Berlin, Germany) using a Cliff-Lorimer method for TEM-EDS investigations and a standardless Peak to Background ratio with Atomic number (Z), Absorption (A), and Fluorescence (F) corrections (P/B ZAF) method for SEM-EDS. To determine the influence of heat treatment on hardness, HV10 tests were conducted using the Zwick Roell (Ulm, Germany) ZHU test device. Tests were conducted on flat, ground, and polished specimens. Five measurements were taken for each variant of heat treatment.

## 3. Results and Discussion

Materials used at high temperatures, to have the longest possible service life, must be characterized by long-term stability of the microstructure, resulting in good creep resistance. The Svante Arrhenius equation can describe diffusion in the solid state (diffusion coefficient):

$$D\left(T\right)=D_{0}\;\times e^{-\frac{Q}{RT}}\;,$$

where D—diffusion coefficient [m^2^/s], D_0_—pre-exponential coefficient [m^2^/s], Q—activation energy of diffusion [kJ/mol], R—gas constant [J/(mol·K)], and T—Temperature [K].

For austenitic steels, the activation energy values of substitutional elements are large. For example, the activation energy of self-diffusion for nickel is 280 kJ/mol, and for tungsten, it is 640 kJ/mol. These values are higher than those for ferritic steels. Therefore, diffusion in austenitic steels occurs more slowly than in ferritic steels. Table 2 shows a comparison of the values of the activation energy of diffusion Q and the value of the pre-exponential factor D_0_ for selected elements in the ferritic matrix and the austenitic matrix.

**Figure 1:**
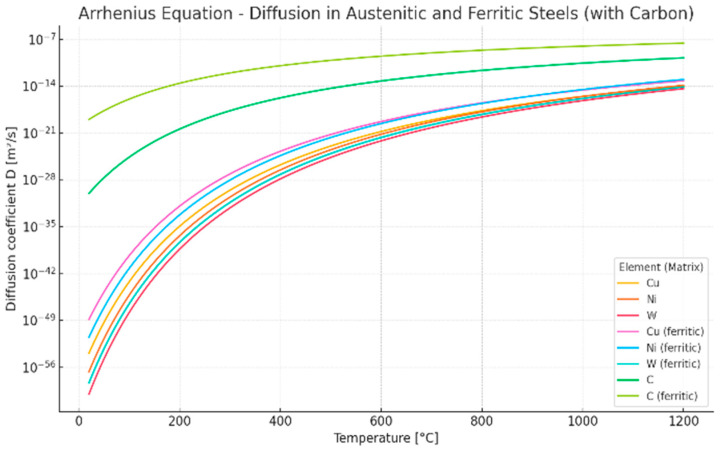
Diffusion coefficient D [m/s] of selected elements in austenitic and ferritic steels.

**Figure 2:**
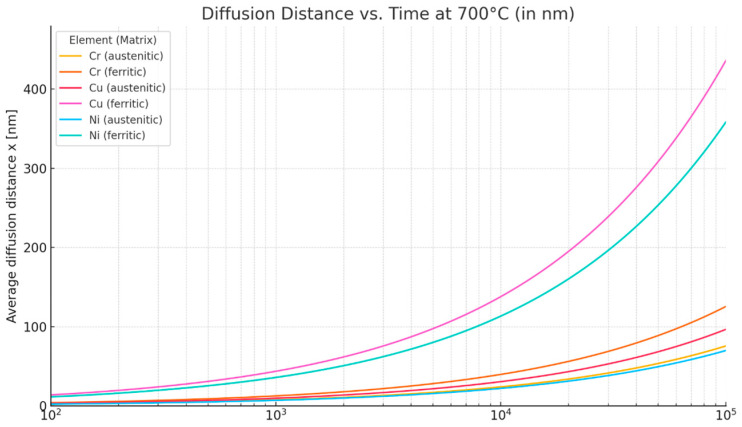
Diffusion distance of selected elements as a function of time in ferritic and austenitic steels.

The long-term stability of the microstructure, and thus the creep resistance of austenitic steels, will generally be higher than that of ferritic steels, which is a consequence of the much slower diffusion rate of substitutional elements in austenite compared to ferrite [35,36,37]. A comparison of the diffusion coefficients of selected substitutional elements (Cu, Ni, W) and C in the austenitic steel matrix and the ferritic steel matrix is shown in Figure 1. Figure 2 shows a comparison of the diffusion paths of selected elements at 700 °C, which corresponds to the temperature of the designed operation of Sanicro 25 steel.

Thanks to its high nickel content (25.35 wt%), the matrix of Sanicro 25 steel is a solid solution austenitic (FCC) structure. The high Cr content (22.5 wt%) provides good oxidation resistance and produces a solution strengthening effect. An additional strengthening effect is supplied by precipitates of M_23_C_6_ carbides that decorate grain boundaries. Sanicro 25 exhibits good resistance to oxidation in the presence of steam at 700 °C, even for extended periods, as confirmed by studies presented in works [17,18]. In the microstructure of this steel, after a certain period of operation or annealing, due to the presence in the composition of this steel of elements such as W (3.4 wt%), Mn (0.5 wt%), Co (1.4 wt%), and Cu (2.98 wt%), there may be precipitates of Sigma, Laves, and Z phases. In addition, precipitates rich in copper, which can cause a precipitate strengthening effect, are also often observed [9,11,38]. Structural components made from Sanicro 25 steel are usually pipes. Their manufacturing process involves many steps, starting with crystallization from the hot phase and ending with the final heat treatment of the finished component. The overall heat treatment carried out will have an impact on the properties of the finished parts. Already entering crystallization from the liquid phase, despite rapid cooling, nucleation and partial growth of precipitates of various phases occur in addition to the austenitic matrix in the material’s microstructure. Some of the precipitates that form during solidification from the liquid phase remain in the material’s microstructure, even after further stages of heat treatment, as is the case with austenitic steels [39,40,41].

### 3.1. Sanicro 25 As-Received CALPHAD Simulation

Authors who study precipitate processes focus on the microstructure of samples after extended periods, as this is of primary importance for the serviceability of these materials, assuming that the presence of precipitates after the supersaturation process is negligible. There are, however, studies on the direction of increasing the strength of austenitic steels through precisely controlled heat treatment [8,9,42,43].

**Figure 3:**
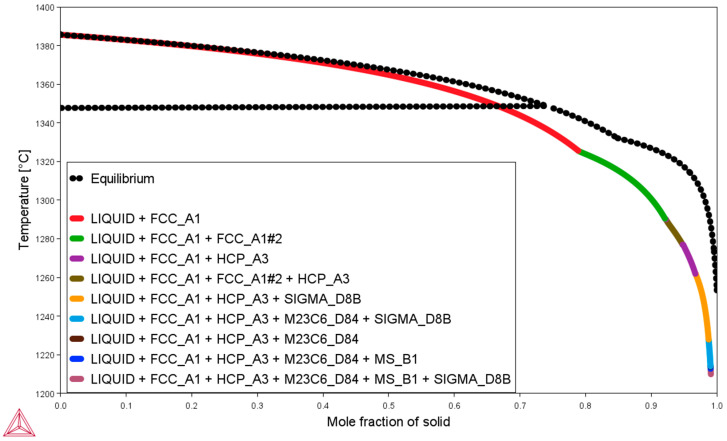
Mole fraction of solid as a function of temperature during the solidification process of Sanicro 25 and the results of Scheil calculations.

The results of the simulation of the crystallization process of Sanicro 25 using the Thermocalc package with the Scheil model are shown in Figure 3. The chemical composition used in the calculations is not the standard chemical composition of Sanicro 25, but rather the composition from the manufacturer’s certificate for a specific product (pipe), which was used in the part of this research on experimental heat treatment processes. The simulation indicates that full crystallization of this steel will occur at 1209 °C. This is much lower than the temperature predicted by Stojan et al. [26] and that of the equilibrium calculations assuming a slow cooling rate. This temperature is very close to the supersaturation temperature during standard heat treatment, annealing after thermomechanical treatment of this steel.

The thermodynamic calculations predict that, after crystallization from the liquid phase, the following matrix phases are present in the microstructure of Sanicro 25: Austenite, a solid solution with an FCC structure; (Cr, Nb)N; precipitates (carbides of the type M_23_C_6_); and the Sigma phase. Thermodynamic calculations predict the remaining phases, but their amount is negligible. The calculated chemical composition of the phases in the final stage of crystallization is presented in Table 3. This composition differs from the equilibrium composition at 1210 °C, as shown in Table 4.

When calculating the crystallization process using the Scheil method, the diffusion processes occurring in the solid state are not taken into account. Therefore, the results presented in the table do not fully correspond to the chemical and phase composition of the material, which is delivered in the as-received state and has undergone complex heat-plastic processing. One of the production stages of the pipe is quite complex, involving hot thermo-plastic processes. In the case of the pipe from which the experimental samples were cut, the final shaping stage is carried out as part of the cold pilgering process. The cold pilgering process produces a texture in the microstructure that remains in the finished pipe, despite the application of solution annealing at 1210 °C for one hour, followed by rapid cooling in the final stage of the process. Cooling processes after heat treatment can be carried out under various conditions, including convection cooling in air and accelerated cooling using forced air. Cooling in water, which provides the highest cooling rate, can also be used. All of the above processes affect the final microstructure, also known as the microstructure in the as-received state. The use of a particular cooling method will therefore affect the cooling rate from the supersaturation temperature, which will consequently affect the microstructure of the component in the delivery state, in this case, the finished pipe. 

**Figure 4:**
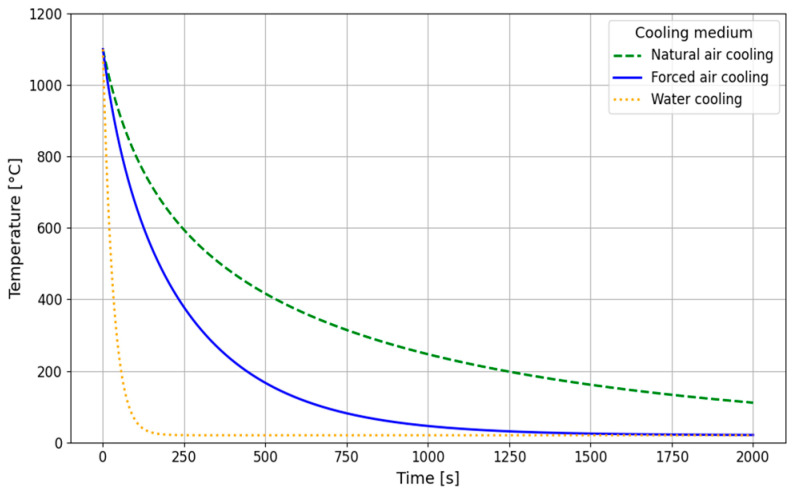
Cooling curves of Sanicro 25 pipe, from a supersaturation temperature of 1210 °C to an ambient temperature of 20 °C, for various cooling methods.

To determine how the cooling rate will affect the microstructure of the finished part, the cooling time of the pipe from supersaturation temperature to room temperature was calculated. The curves of temperature changes during cooling, of a pipe with a length of 1 m, an outer diameter of 38 mm and an inner wall thickness of 9 mm (the dimensions of the pipe used in the experiment) are shown in Figure 4. For the calculations, the density of Sanicro 25 steel was assumed to be 8.32 g/cm^3^. Linear changes in specific heat were considered over the analyzed temperature range, resulting in particular heats of 470 J/kgK at 20 °C and 665 J/kgK at 1100 °C, respectively. It was believed that cooling of the sample in air after the supersaturation process occurs by convection and radiation. For the calculations, the heat transfer coefficient by convection was considered to be 20 W/(m^2^/K) for air, 100 W/(m^2^/K) for air blowing, and 1000 W/(m^2^/K) for cooling in water. A steel surface emissivity of 0.7 was assumed [44,45]. Temperature changes were determined based on the following equation:

$$\frac{dT}{dt}=\frac{1}{m\cdot c\left(T\right)}[h\cdot A\cdot \left(T-T_{ambient}\right)+\varepsilon \cdot k\cdot A\cdot (T^{4}-T_{ambient}^{4})]$$

where:

ε—emissivity coefficient

k—Stefan–Boltzmann constant [W/(m^2^·K^4^)]

h—heat transfer coefficient [W/(m^2^·K)]

m—mass—a pipe with a length of 1 m, an outer diameter of 38 mm, and an inner wall thickness of 9 mm [kg]

A—heat transfer surface (outer surface of the pipe) [m^2^]

T—temperature [K]

If the pipe is cooled from a supersaturation temperature of 1210 °C, the temperature of 200 °C (at which the diffusion rate is, as far as precipitation processes are concerned, practically negligible) will be reached after 54 s in the case of cooling in water and about 1200 s in the case of cooling in air.

**Figure 5:**
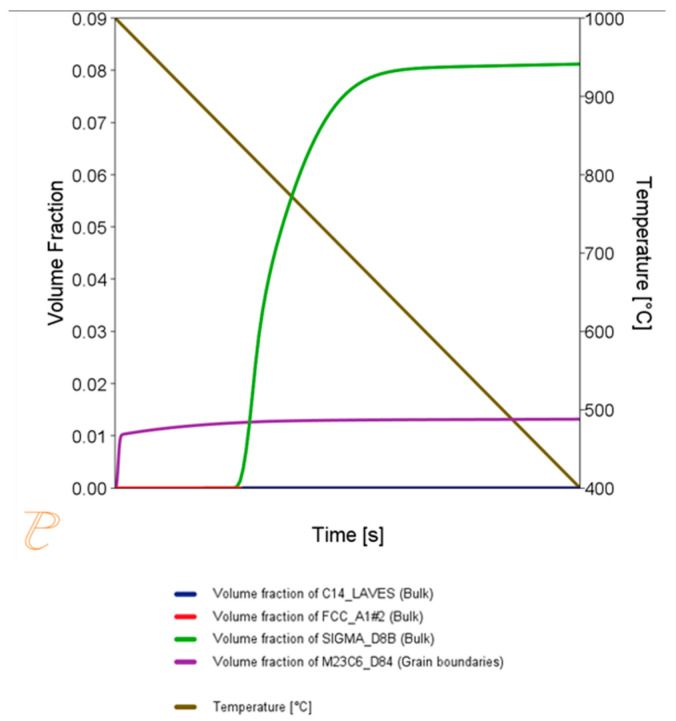
Changes in the phase contribution of phases precipitated during the cooling process in air after the supersaturation process. Phases that can precipitate: Sigma and M_23_C_6_ carbides.

During the cooling process, phase transformations can occur, involving precipitation processes from the solid solution. Thermodynamic calculations for this process were performed using the Thermo-Calc program’s Prisma module, with the previously calculated temperature profile used for the thermodynamic simulations. The chemical composition of the material provided with the manufacturer’s certificate (melt composition) was taken as the composition of Sanicro 25, with the understanding that, in reality, the matrix will show slight differences due to the presence of a small number of primary precipitates formed during crystallization from the solid phase and thermo-plastic processes during the manufacture of the pipe. Using the Prisma package, the precipitation processes occurring during cooling in free air were simulated. During cooling of this type, the time to reach 400 °C is approximately 411 s. The simulation results of the precipitation processes are shown in Figure 5. The simulation predicts the precipitation of Sigma phase and M_23_C_6_-type carbides from the matrix. In practice, the heat treatment procedure that is used after the supersaturation process is quenching in water. Cooling in water is much faster than cooling in air. Calculations show that a pipe made of Sanicro steel cooled in water will reach a temperature of 400 °C in about 21 s. Precipitation processes during such rapid cooling will occur to a very limited extent.

### 3.2. Sanicro 25 As-Received, Microstructure

**Figure 6:**
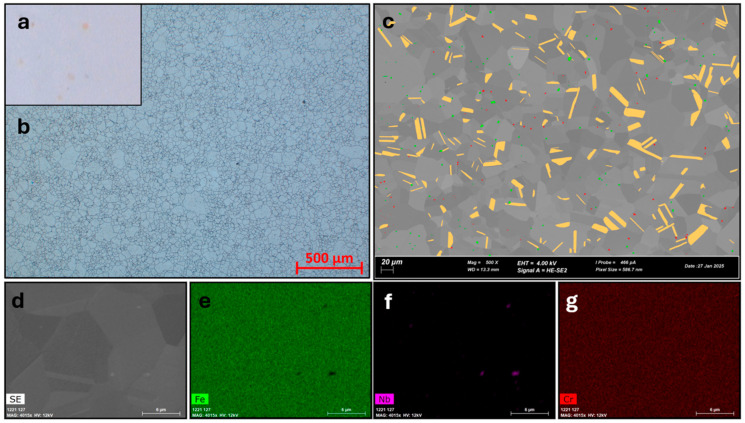
Microstructure of the material in the as-delivered state. (**a**) The microstructure observed using BF-LM, showing characteristic contrast, golden color originating from nitrides. (**b**) Nitride precipitates are marked in red (LM). (**c**) Shows the microstructure of the sample observed using SEM. Carbonitride precipitates are marked in red, while other primary precipitates are marked in green. Equiaxed grains and a significant number of twins are observed in the microstructure. Twin areas are schematically marked in a cream color. (**d**–**g**) Show maps of the distribution of selected elements (Fe, Nb, Cr). A uniform chemical composition and areas enriched in Nb are visible.

The microstructure of the material in the delivery state (after supersaturation) is shown in Figure 6.

**Figure 7:**
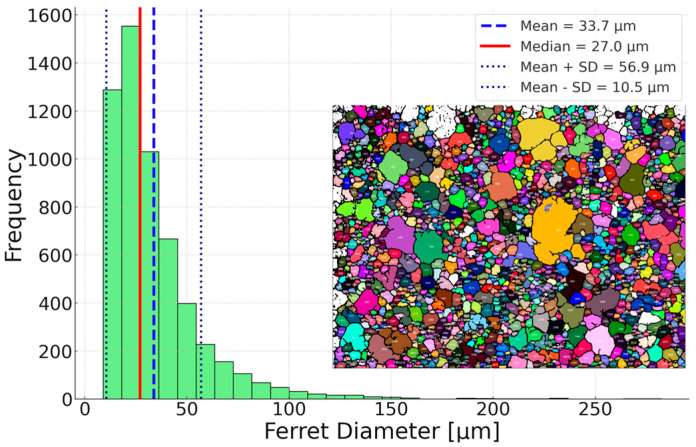
Histogram of Ferret diameter frequency for material in as-received state, average and mean grain size with standard deviation of the mean different colors represent different grains.

The steel in its as-received condition exhibits grains of varying sizes, with a few larger grains observed, while the vast majority of grains are small. The average grain size is 33.7 µm, with a standard deviation of 23 µm. A histogram illustrating the distribution of grain size, along with the average and mean grain sizes, is presented in Figure 7. The observations made, including the distribution maps of the chemical composition produced by the SEM-EDS method, do not allow the presence of precipitates to be confirmed unequivocally.

**Figure 8:**
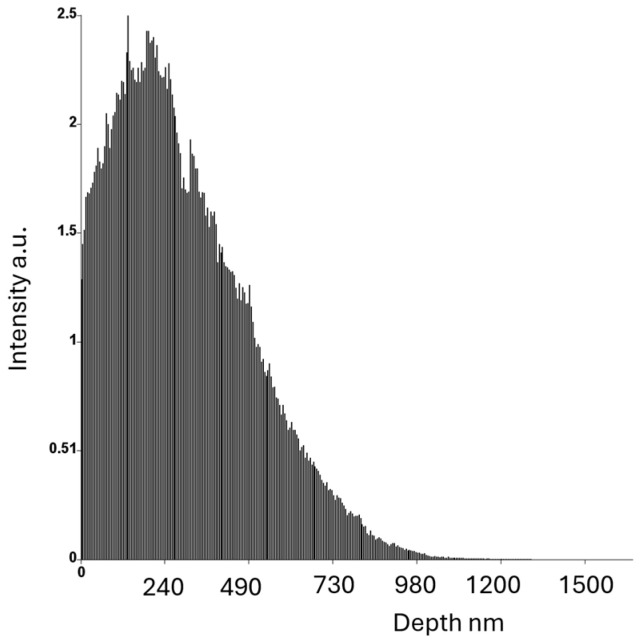
Histogram of X-ray Fe Kα as a function of depth and the results of Casino simulations.

The studies, especially those performed in the SEM, are derived from the volume of the material due to the significant area of interaction of the beam with the volume of the analyzed material. When materials with a significant iron content are observed at an accelerating voltage of 20 kV, the K_α_ characteristic radiation originates from a depth of more than 240 nm, as shown in Figure 8.

**Figure 9:**
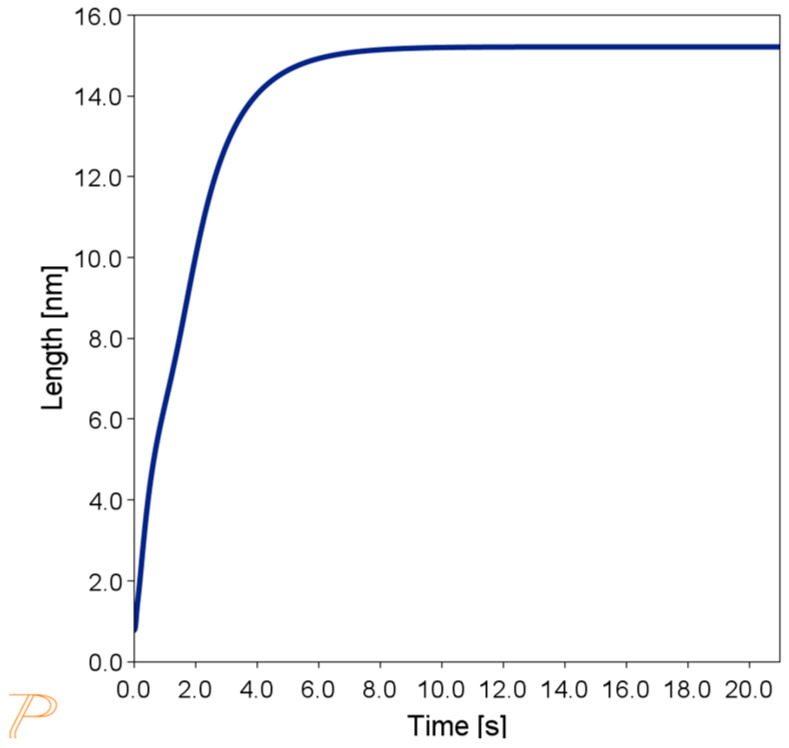
Size of M_23_C_6_-type precipitates at grain boundaries during cooling from supersaturation temperature in water.

**Figure 10:**
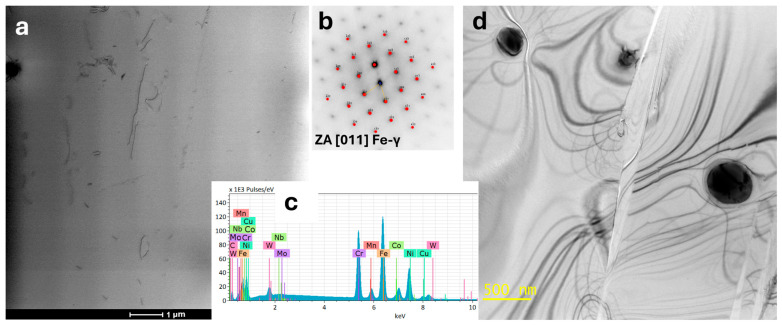
Microstructure in the as-received state observed by TEM. Visible dislocations (**a**) in the austenitic matrix with ZA orientation [011], (**b**) electron diffraction pattern from the matrix with a simulated diffraction pattern superimposed, (**c**) TEM-EDS spectrum from the matrix area, and (**d**) single primary precipitates observed within the matrix (TEM-BF).

The simulation results show that these precipitates can form within the first 6 s of cooling and reach a size of approximately 15 nm (Figure 9). Therefore, identification of nanometric carbides by SEM-EDS will not be possible. The microstructure of the sample, as observed by TEM, is shown in Figure 10. The microstructure consists of an authentic FCC matrix with a negligible amount of dislocations and single precipitates. Additionally, there is a contrast in the grain boundary areas in the BF-TEM mode, which can originate from grain boundaries or precipitates of very small sizes. Simulation studies of Sanicro 25 after rapid cooling in water suggest the presence of M_23_C_6_-type carbide precipitates in addition to the matrix. Precipitates of this type can already appear at the boundaries between grains during rapid cooling from the supersaturation temperature. 

The microstructural studies shown in Figure 10 did not allow us to confirm the presence of these precipitates, likely due to their very small size, which can be located at the grain boundaries of the austenitic matrix. Precipitates of this size located at the grain boundaries will also be difficult to observe by transmission microscopy techniques and microscopy techniques in general due to the strong stress contrast and diffraction contrast changes occurring in these areas as a consequence of the different orientation of adjacent grains. An additional difficulty in observation is that the size of the carbide precipitates is much smaller than the thickness of the lamellae for TEM studies. The lamella thickness was estimated by zero-loss peak estimation using an Electron Energy Loss Spectroscopy (EELS) to be about 100 nm. The results of simulation studies on the cooling process in water correlate well with the observations of the microstructure of the tested steel in its as-delivered state, except that precipitates of M_23_C_6_ carbides could not be observed.

**Figure 11:**
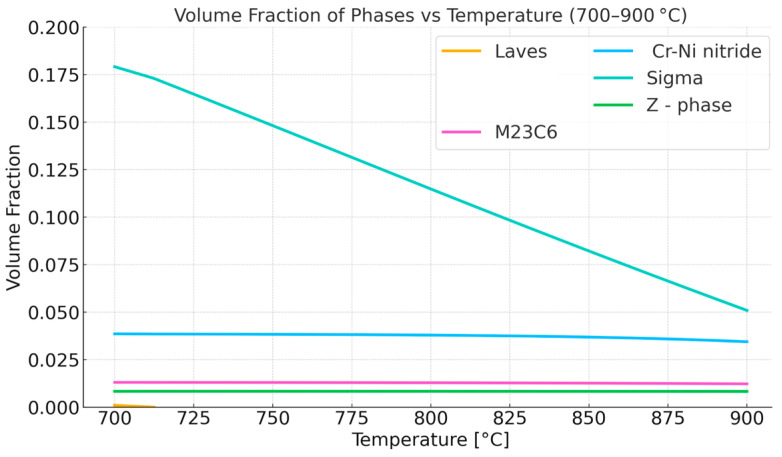
Equilibrium phase composition in the temperature range of 700–900 °C. Matrix excluded.

The next step was to conduct an experimental study to investigate the initial stages of changes in the microstructure of Sanicro 25 within the temperature range of 700 °C to 900 °C. The temperature of 700 °C corresponds to the expected operating temperature of this steel, e.g., in a secondary steam superheater, while the higher temperatures are already beyond the currently predicted long-term (100,000 h) operating temperatures of this steel. Before the heat treatment experiment, CALPHAD, equilibrium simulations were performed in the temperature range 700–900 °C. Figure 11 illustrates the equilibrium phases that occur in the temperature range of 700–900 °C. The same phases occur in the analyzed temperature range. The primary difference is the decreasing contribution of the Sigma phase with increasing temperature.

Thermodynamic simulations predict the disappearance of the Laves phase slightly above 700 °C. The most significant change is in the volume fraction of the intermetallic Cr-rich and Mo-rich Sigma phase, decreasing from 17.5% at 700 °C to 5% at 900 °C. The chemical composition of the Sigma phase also changes. As the temperature increases, the W content increases while the Cr and Fe content decreases.

**Figure 12:**
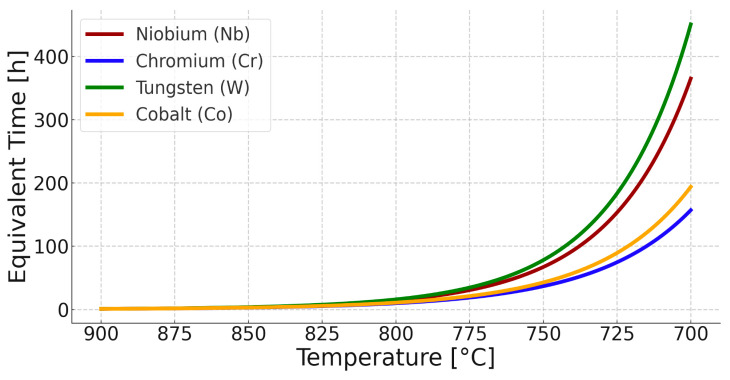
Time equivalent, related to the temperature of 900 °C, assuming that the diffusion processes of substitutional elements control the processes of precipitation and phase transformation.

**Figure 13:**
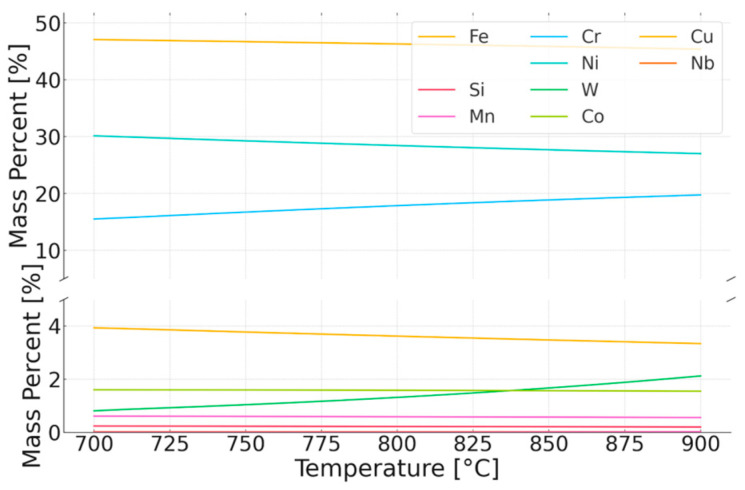
Equilibrium chemical composition of the matrix in the temperature range of 700–900 °C.

**Figure 14:**
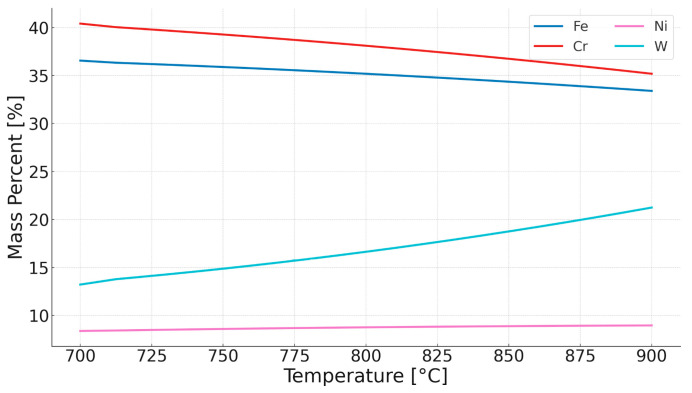
Equilibrium chemical composition of the Sigma phase in the tested steel in the temperature range of 700–900 °C.

Thus, there is an increase in the equilibrium concentration of Cr in the matrix. Thermodynamic calculations under equilibrium conditions at 700 °C (the proposed application temperature for this steel) predict the existence of volume fractions of the following phases: 76% austenitic matrix (FCC), 13% Sigma phase, 3.7% (CrFeNi)N; 1.3% M_23_C_6_ carbides, and small amounts (<0.001%) of the Laves phase and Z phase. Studies on the microstructure and phase composition of this steel after prolonged annealing and oxidation times are presented in papers [17,18]. The experimental studies consisted of annealing samples made of Sanicro 25 with dimensions (about 10 × 10 × 9 mm) in a furnace heated to temperatures of 750, 800, 850, and 900 °C in an air atmosphere. The purpose of both the simulation study and the actual experiment was to determine the changes in the microstructure of the steel in the initial stages of operation and during annealing at a temperature range 200 °C higher than the recommended temperature. Assuming that the determinant of phase transformations and their rate is the diffusion of substitutional elements, it is possible to estimate how raising the temperature will affect the time of occurrence of phase transformations—the processes of precipitation of thermodynamically stable phases at a given temperature. Based on the equation presented in the earlier part of this work, calculations were carried out to determine the change in time as a function of temperature. Figure 12 illustrates the time equivalent at a temperature of 900 °C. One hour of annealing at 900 °C corresponds to about 157 h of annealing at 700 °C for chromium and about 450 h for tungsten, due to the much larger atomic radius of this element. The above assumptions do not account for the precipitation processes or the resulting changes in the chemical composition of the matrix that occur under actual heat treatment conditions, as shown in Figure 11, Figure 13 and Figure 14. They are therefore only a rough description of the microstructure evolution processes occurring under real conditions.

### 3.3. Simulations of the Precipitation Process Using the Thermo-Calc Package

**Figure 15:**
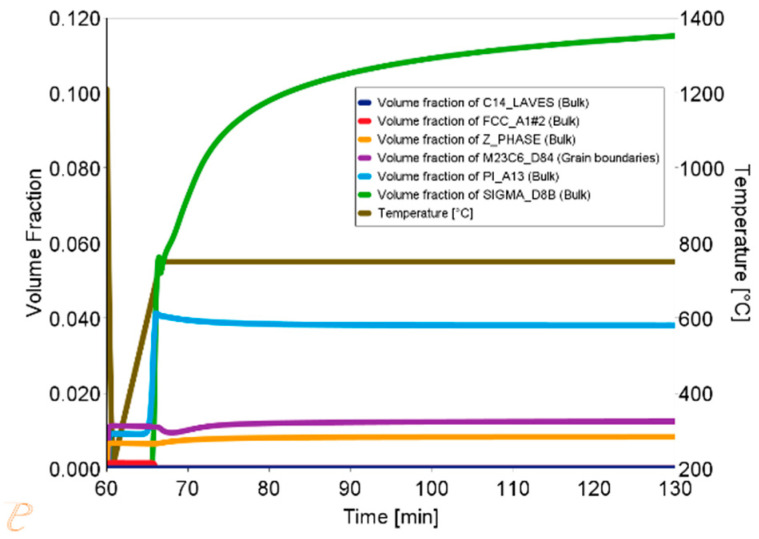
Volume fraction of phases during the annealing process at 750 °C for 1 h, preceded by annealing at 1210 °C for 1 h.

Thermodynamic simulations were performed using the Thermo-Calc software package, version 2025a, and two thermodynamic databases specifically designed for steels, TCFe10 and MobFe5. The simulation focused on calculating phase fractions. The phases for simulation were selected based on previously performed equilibrium calculations. The simulations also determined the precipitate sizes and changes in the matrix chemical composition during annealing at 750, 800, 850, and 900 °C. It was assumed that the steel is held at 1210 °C for one hour and then cooled to the target temperature, where it is held for 60 min. The results of the simulation at 750 °C are shown in Figure 15.

**Figure 16:**
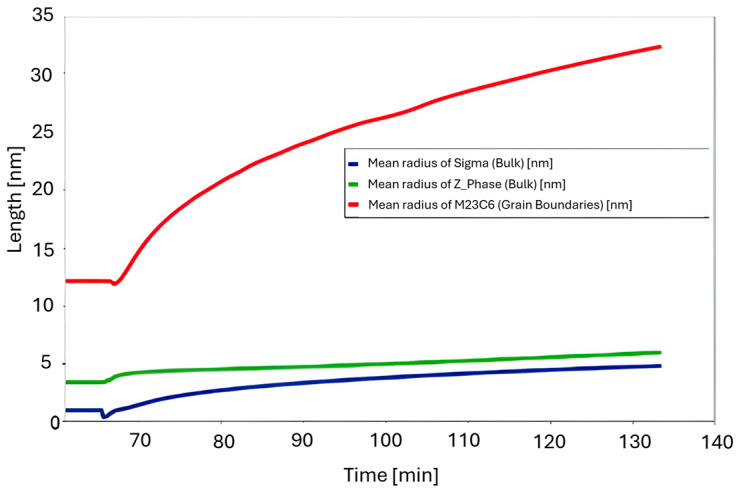
Change in precipitate size during the annealing process at 750 °C for one hour, preceded by annealing at 1210 °C for 1 h.

**Figure 17:**
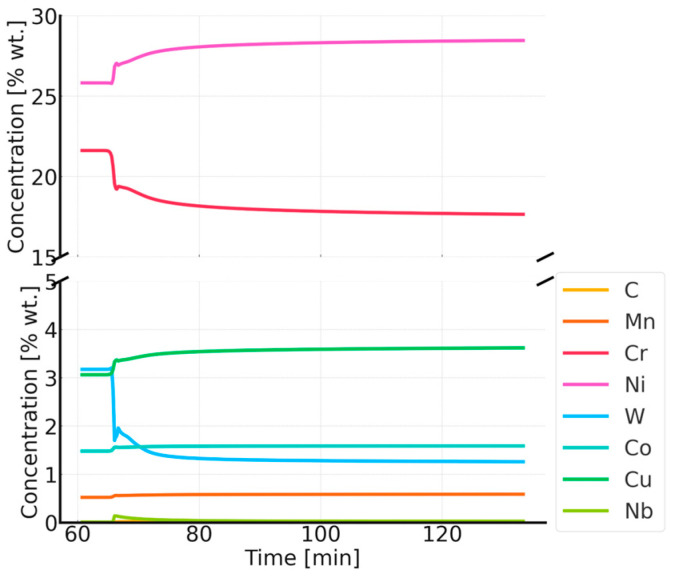
Changes in the chemical composition of the matrix during annealing after supersaturation. Annealing for one hour at 750 °C, preceded by annealing at 1210 °C for 1 h.

The simulation results for the phase composition and the change in precipitate size are shown in Figure 16 and Figure 17. The thermodynamic simulations carried out show that the main phases, such as the Sigma phase and (CrNiFe)N, will precipitate from the matrix, considering only the volume fraction. The amount of the Sigma phase will increase during annealing. The average size of precipitates will be very small, ranging from 30 nm for M_23_C_6_ carbides to 5 nm for other precipitated phases (Figure 16). The results of the simulation of the change in the chemical composition of the matrix during annealing at 750 °C are shown in Figure 17.

**Figure 18:**
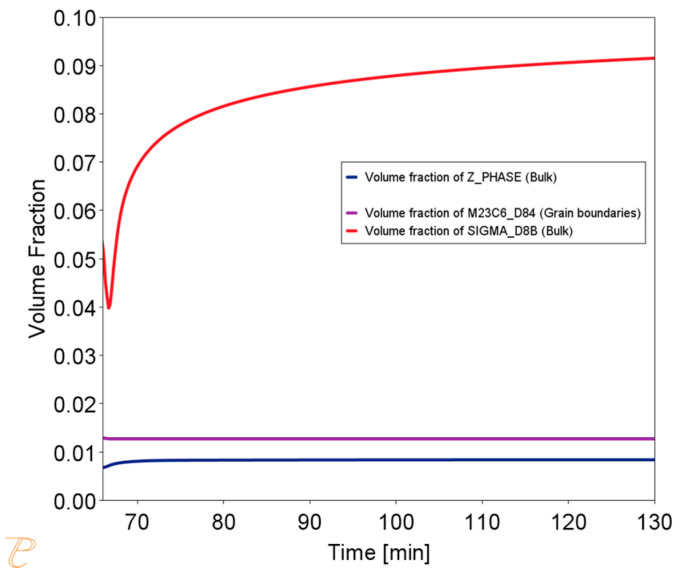
Volume fraction of phases during the Sanicro 25 annealing process at 800 °C for 1 h, preceded by annealing at 1210 °C for 1 h.

**Figure 19:**
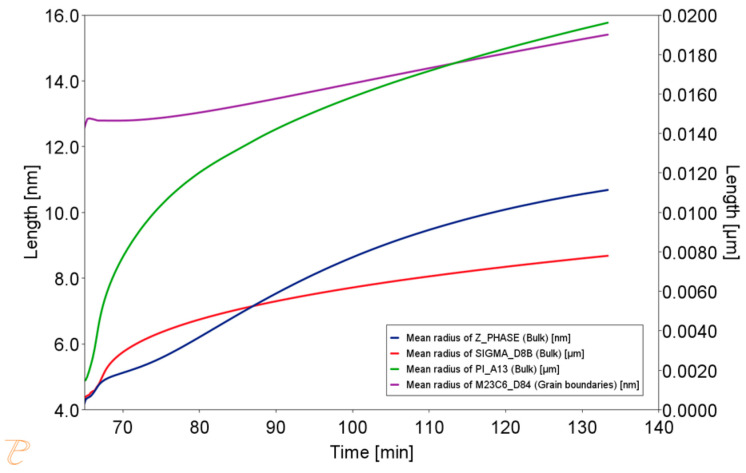
Change in the size of precipitates during the annealing process at 800 °C for 1 h, preceded by annealing at 1210 °C for 1 h.

**Figure 20:**
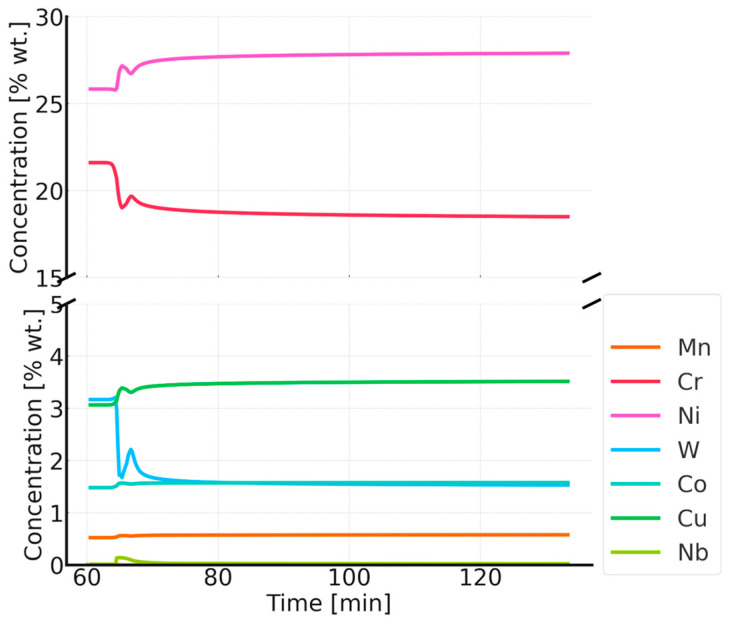
Changes in the chemical composition of the matrix during annealing after supersaturation. Annealing for 1 h at 800 °C, preceded by annealing at 1210 °C for 1 h.

Thermodynamic simulations performed at 800 °C (Figure 18, Figure 19 and Figure 20) predict depletion of the matrix in Cr, as well as nucleation of the Sigma phase, Z phase, and carbides. The predicted particle sizes are larger than for annealing at 750 °C. The predicted precipitate size is smaller than that observed in reality, especially for carbide precipitates at the grain boundaries.

**Figure 21:**
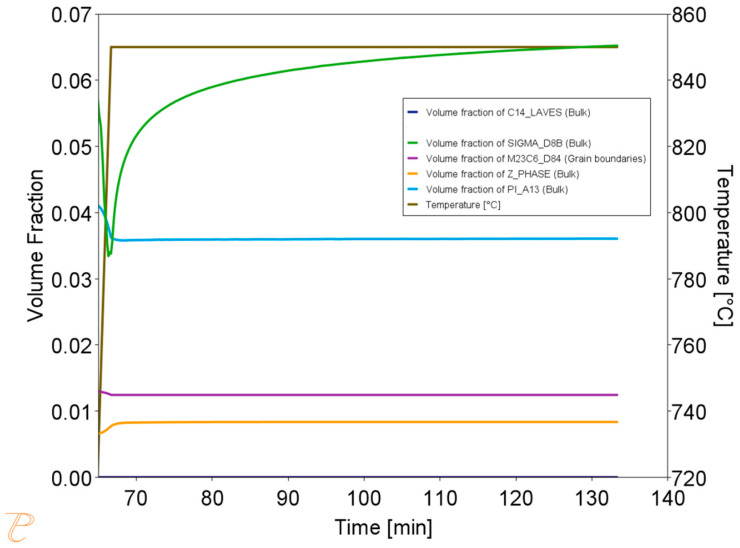
Volume fraction of phases during the Sanicro 25 annealing process at 850 °C for 1 h, preceded by annealing at 1210 °C for 1 h.

**Figure 22:**
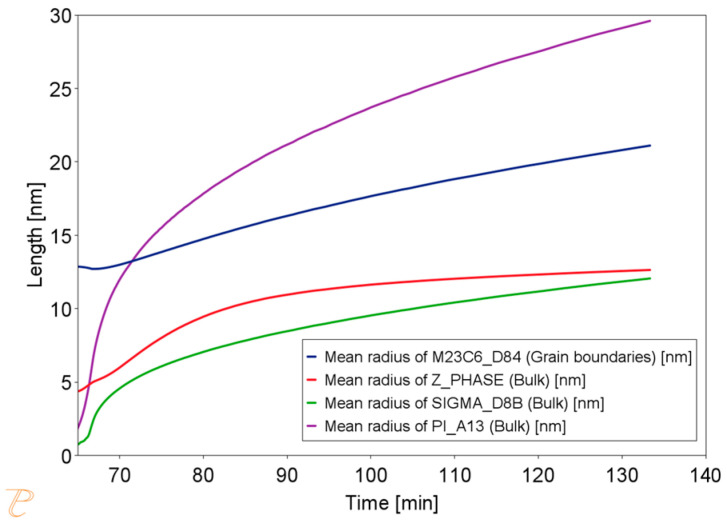
Change in the precipitate size during the annealing process at 850 °C for 1 h, preceded by annealing at 1210 °C for 1 h.

**Figure 23:**
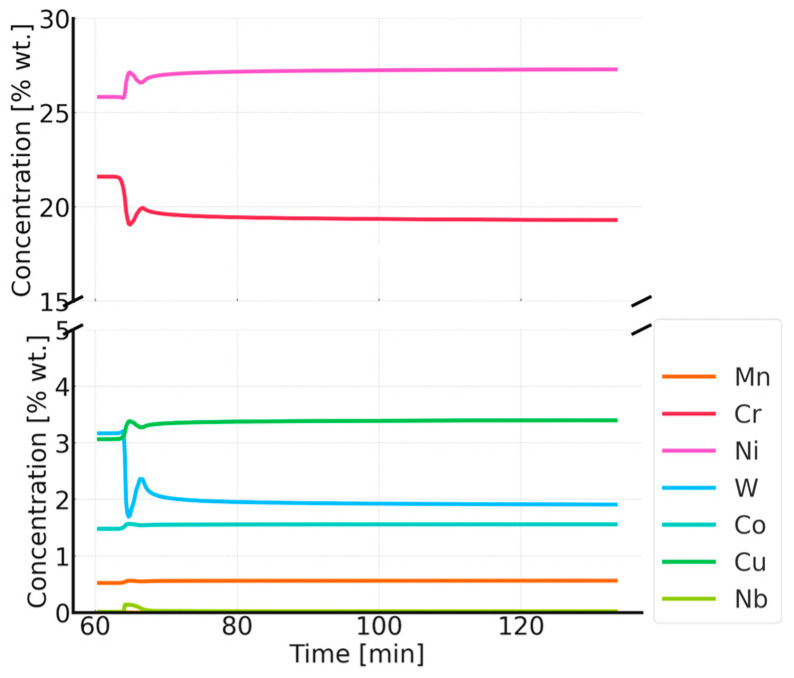
Changes in the chemical composition of the matrix during annealing after supersaturation. Annealing for 1 h at 850 °C, preceded by annealing at 1210 °C for 1 h.

Results of the simulation of the annealing process at 850 °C for 1 h, preceded by annealing at 1210 °C for 1 h, are presented in Figure 21, Figure 22 and Figure 23. The chemical composition of the matrix after annealing at 850 °C, as far as Cr content is concerned, is more similar to the nominal chemical composition of Sanicro 25, suggesting that there is a smaller amount (volume of precipitates rich in this element). On the other hand, in the initial period of annealing, there is a decrease in the W content in the matrix, due to the precipitate of W-enriched phases, which may suggest the precipitation of Laves phases, or the depletion of carbides in Cr and enrichment in W. The size of precipitates is much larger, especially in the case of the NbN phase; the simulation result corresponds quite well to the trends observed in the actual microstructure of the material.

**Figure 24:**
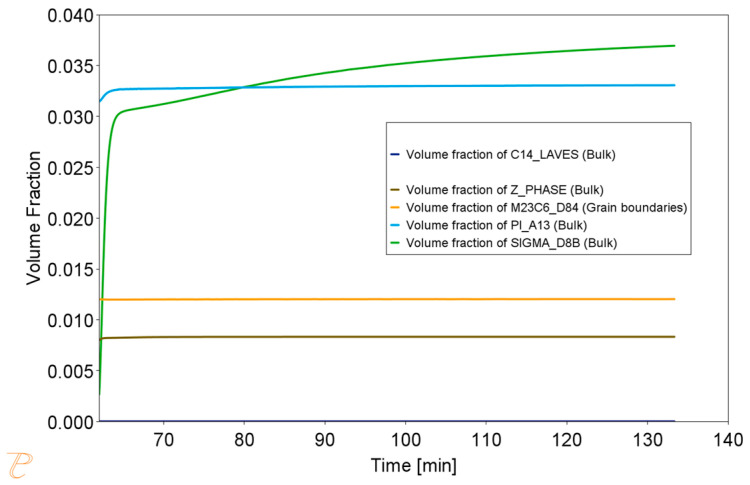
Volume fraction of phases during the Sanicro 25 annealing process at 900 °C for 1 h, preceded by annealing at 1210 °C for 1 h.

**Figure 25:**
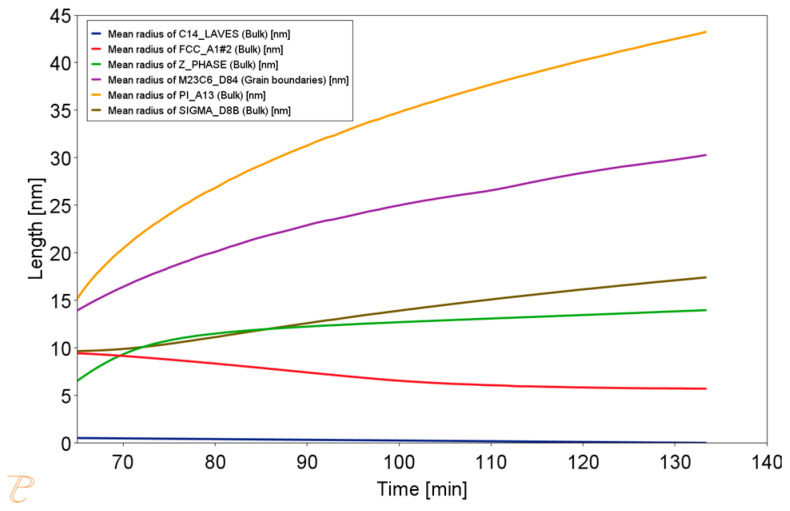
Change in the size of precipitates during the annealing process at 900 °C for 1 h, preceded by annealing at 1210 °C for 1 h.

**Figure 26:**
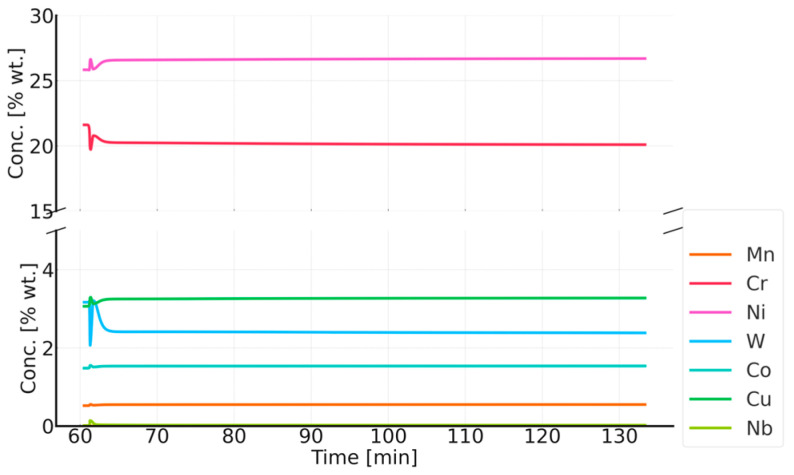
Changes in the chemical composition of the matrix during annealing after supersaturation. Annealing for 1 h at 900 °C, preceded by annealing at 1210 °C for 1 h.

The results of thermodynamic simulations for annealing at 900 °C are presented in Figure 24, Figure 25 and Figure 26. The phase changes predicted by the simulations are the largest in this case. The simulation also predicts the largest size of precipitates, which is also consistent with the observations.

**Figure 27:**
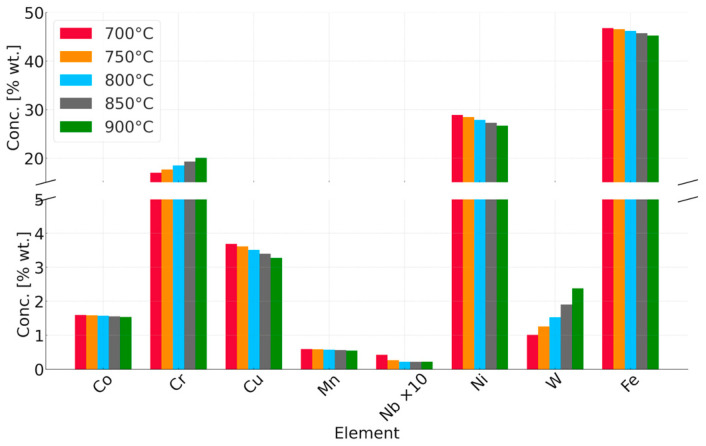
Simulation results of the chemical composition of the matrix after the annealing process, depending on the annealing temperature.

In the case of annealing at 900 °C, the simulations predict changes in the chemical composition of the matrix during the initial stage of heating. Precipitation processes occur quite quickly, and the chemical composition of the matrix stabilizes, approaching the nominal chemical composition of Sanicro 25. The predicted chemical composition of the matrix after the annealing process for all temperatures is shown in Figure 27.

As the annealing temperature increases, the Cr and W content in the matrix increases, while the Cu content decreases. Simulations show that during the annealing process, there is an intensive precipitation of the Sigma phase, especially at 750 °C. The carbide precipitates observed at the boundaries are predicted by simulations over the full temperature range at which the experiment was conducted. At low temperatures, however, these precipitates are small in size (a few to several nm), making them difficult to observe using electron microscopy techniques. The phase precipitates nucleate preferentially at dislocations and grain boundaries, which is typical of austenitic steels.

### 3.4. Investigation of the Microstructural Changes After Annealing

**Figure 28:**
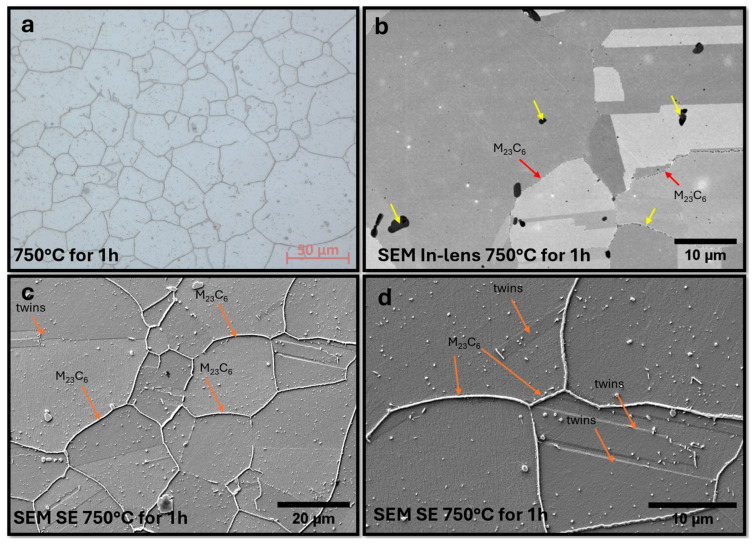
Microstructure of Sanicro 25 after annealing for one hour at 750 °C. LM (**a**), SEM un-etched sample, in-lens detector, visible primary precipitates and precipitates at grain boundaries. (**b**) SEM etched sample, visible precipitates at boundaries and minor precipitations within grains. (**c**,**d**) SEM sample observed at higher magnifications.

The annealing process, conducted for one hour at 750 °C, resulted in significant changes to the material’s microstructure. These are shown in Figure 28.

After only a short annealing time in the steel, precipitates can be observed at the boundaries; these are M_23_C_6_-type carbide precipitates. Precipitates of this type are difficult to observe in the SEM, particularly in the case of the unannealed sample used for EDS testing. By using a low accelerating voltage of 4 kV and an in-lens detector, it was possible to make observations. M_23_C_6_ precipitates were marked with a red arrow, while mostly fine precipitates were observed inside the grains. The presence of the precipitates was confirmed on the etched sheets. In addition to the M_23_C_6_ precipitates, there were other precipitates, probably of the NbN phase, the presence of which will increase the strength of this steel. Precise determination of the type of precipitates using SEM-EDS was not possible due to the large volume of interacting electrons and the area of emission of characteristic X-rays, much larger than the size of the precipitates, so that the chemical composition was blurred. Thermodynamic simulations showed that the main phases, such as the Sigma phase and (CrNiFe)N, will precipitate from the matrix. At the same time, only the volume fraction of the Sigma phase will increase during annealing. On the other hand, the average size of precipitates will be very small in the range up to 30 nm for M_23_C_6_ carbides and 5 nm for other precipitated phases.

The simulation predicts depletion of Cr in the matrix, which could not be confirmed experimentally; the Cr content in the matrix decreases from the nominal value to approximately 18 wt%. SEM-EDS studies indicate that areas of the matrix contain Cr in an amount slightly less than the nominal content; however, the area of interaction of the beam for electrons at 20 kV is greater than 1 µm. The application of a high accelerating voltage is necessary to ionize elements with high atomic numbers present in Sanicro 25. SEM-EDS analysis using low voltages and identification based on characteristic radiation of the Lα energy range is practically infeasible due to the large number of elements and similar radiation values, as well as the difficulty in energy deconvolution for the elements in the chemical composition of Sanicro 25.

**Figure 29:**
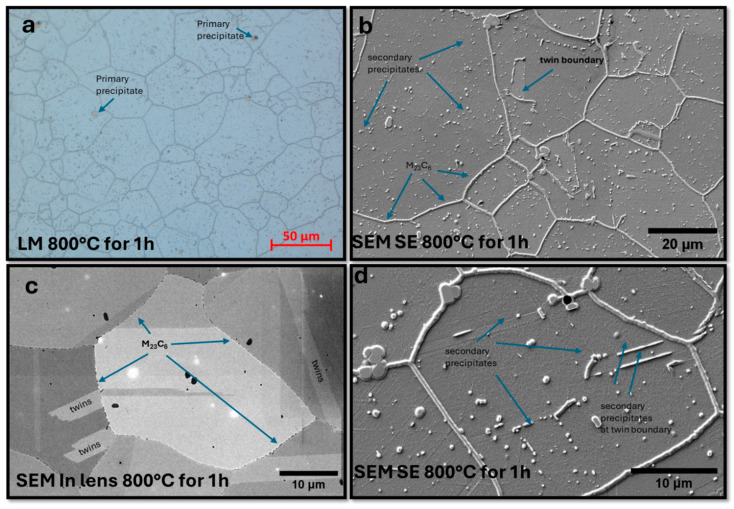
Microstructure of Sanicro 25 sample annealed at 800 °C for one hour. (**a**) LM: grain boundaries and primary precipitates, (**b**) SEM SE: M_23_C_6_ carbides on the etched sample at the boundaries and secondary precipitates inside the grains, (**c**) SEM in-lens: visible primary precipitates and isolated carbides at the grain boundaries, (**d**) SEM SE: visible secondary precipitates on the twins inside the grain boundaries.

The microstructure of the sample after annealing at a higher temperature, 800 °C, is shown in Figure 29. Increasing the annealing temperature resulted in an intensification of the precipitation processes. Observations in the SEM highlighted isolated carbide precipitates of the M_23_C_6_ type at grain boundaries and secondary precipitates within the grains. The use of HR-SEM allowed the observation of secondary precipitates on twin boundaries and also on dislocation structures inside the grains. Grains with spherical morphology and elongated grains were observed, especially at twin boundaries.

**Figure 30:**
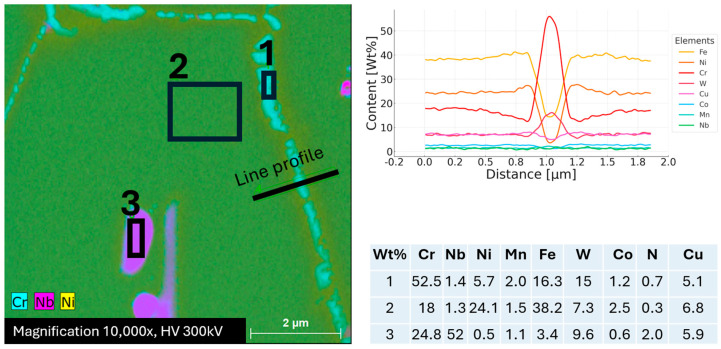
Map of chemical composition distribution with marked STEM-EDS analysis points (1–3) and linear profile from the area of precipitates at the grain boundary.

**Figure 31:**
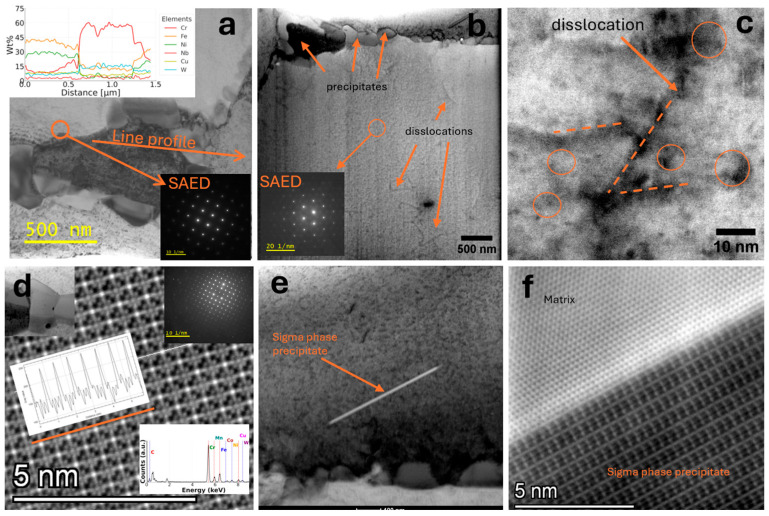
Microstructure of Sanicro 25 after supersaturation and annealing at 800 °C for 1 h. Cr-rich carbide precipitates observed at grain boundaries, linear profile of chemical composition distribution (**a**). SAED from the carbide area, matrix and carbide precipitates at boundaries, SAED from the matrix (**b**). Contrast variations (ovals) around dislocations (dash-lines) and precipitates observed in STEM-BF at high magnification (**c**). HRSTEM-HAADF from the carbide area, SAED from this area, EDS spectrum and STEM- HAADF intensity profile from the location of the observed atomic columns (**d**). Sigma phase precipitate in the matrix (**e**). HRSTEM-HAADF image of the phase boundary between the matrix and the Sigma phase (**f**).

Microstructure studies of this sample were also performed using TEM. The result of the analysis using the STEM-EDS is shown in Figure 30 and Figure 31. At grain boundaries, there are precipitates rich in Cr, and they are also enriched in W. In high-alloy steels such as Sanicro 25, heat treatments or exposure to elevated temperatures, as during this study, can lead to the formation of precipitates at grain boundaries, typically Cr-rich carbides like M_23_C_6_. These precipitates preferentially form at grain boundaries because these areas have higher free energy compared to the grain interiors, making them favorable sites for the nucleation of M_23_C_6_ carbides. The formation of Cr-rich precipitates results in local compositional changes within the matrix, creating a region adjacent to the precipitates—the depletion zone. This zone is characterized by a decrease in chromium content due to the diffusion of chromium into the forming carbides. For the investigated sample, there was a depletion of approximately five wt.% chromium within a region of about 500 nm from the center of the grain boundary, indicating significant diffusion of chromium atoms to the precipitates. This localized depletion can affect material properties, leading to reduced corrosion resistance [46,47], altered microstructural stability, and changes in mechanical behavior, including decreased creep resistance. At the same time, the local concentration of nickel and iron increases within this depletion zone because these elements do not directly participate in the formation of chromium-rich carbides. As chromium migrates preferentially to the precipitates, the relative concentrations of Ni and Fe rise in these depleted areas. Beyond approximately 700 nm from the center of the grain boundary, the chemical composition stabilizes and returns to the typical levels of Sanicro 25 matrix. This indicates that, past this point, the effects of diffusion are significantly reduced or negligible, and the microstructure remains relatively unaffected. A detailed phase analysis was carried out using TEM. The results are shown in Figure 31.

**Figure 32:**
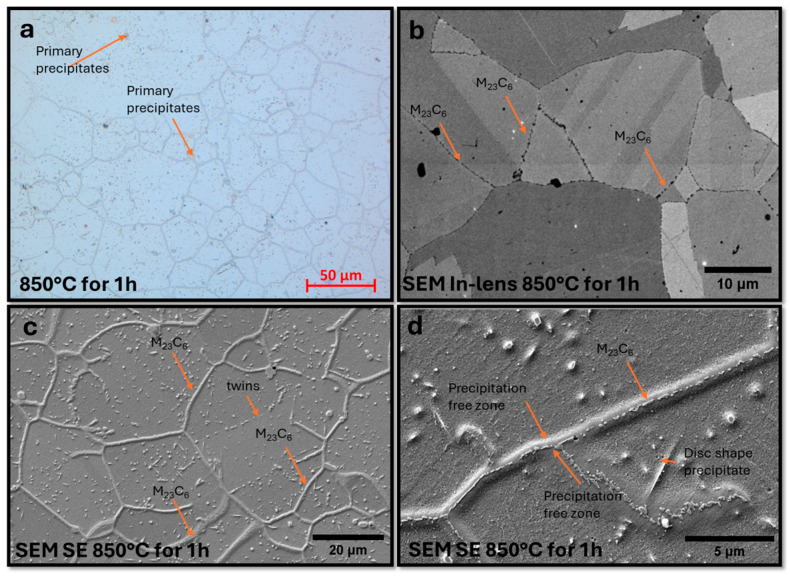
Microstructure of Sanicro 25 samples annealed at 850 °C. LM: primary precipitates and precipitates at grain boundaries (**a**). SEM: M_23_C_6_ precipitates at grain boundaries and precipitates inside grains, areas free of precipitates and plate/disc-shaped precipitates (**b**–**d**).

Precipitates at grain boundaries are visible in the micrograph; these precipitates were identified as M_23_C_6_-type carbides based on electron diffraction and STEM-EDS chemical composition analysis, as shown in Figure 31a. A significant number of dislocations were observed within the grains, shown as lines in Figure 31b. SAED confirmed the FCC matrix structure; the carbide precipitates at the boundaries do not show coherence with the matrix. At higher magnification, precipitates in dislocation areas were imaged in Figure 31c. Figure 31d shows precipitates imaged using the STEM-HAADF technique, for which the contrast is proportional to the atomic number Z [27]. Therefore, it is possible to determine from the image which atomic columns contain heavier elements. The high-resolution image is of a precipitate. The plot shows a linear profile of the intensity distribution along one plane of this crystal. SAED and chemical composition analysis were also performed in this area (Figure 31d). A precipitate with a different morphology—a lamellar precipitate—was observed. Such a precipitate is shown in Figure 31e. At high magnification (Figure 31f), the boundary of the epsilon phase precipitate with the matrix is visible, exhibiting partial coherence. This precipitate is elongated in one plane, characterized by a preferred growth direction, due to the nature of the stresses around this precipitate. Precipitates were observed in the microstructure of Sanicro 25 after annealing at 850 °C (Figure 32a), and carbide precipitations were observed during the observation of the unetched sample (Figure 32b).

**Figure 33:**
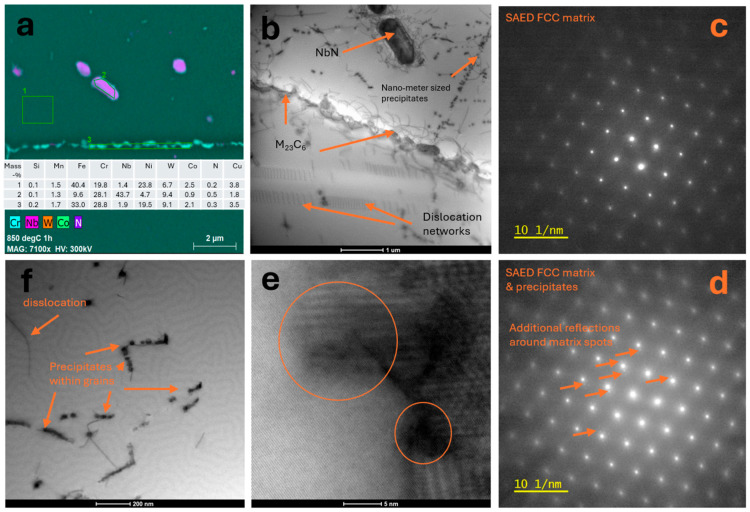
Microstructure of Sanicro 25 annealed for 1 h at 850 °C, observed by TEM, map of chemical composition distribution of elements in the grain boundary area, and results of STEM-EDS analysis for the regions marked as 1,2 and 3 (**a**). Microstructure observed in BF-TEM, visible fine precipitates at the grain boundary, larger NbN precipitates (**b**). SAED from the matrix (**c**) and SAED from the matrix with precipitates, visible additional reflections around the matrix reflections (**d**). visible dislocations, and precipitation nucleation on dislocations (**e**,**f**).

The number of precipitates inside grains is significantly larger compared to the annealed beads at lower temperatures (Figure 32c); the disk-shaped precipitates have substantially larger dimensions. There are precipitate-free areas around precipitates at the boundaries, probably due to the consumption of the Cr reservoir during the formation of M_23_C_6_ precipitates (Figure 32d). Observations with STEM-EDS revealed M_23_C_6_ carbides enriched in tungsten and the occurrence of larger NbNW precipitates (Figure 33a)

The structure also exhibits very fine, nanometer-sized precipitates that nucleate on privileged nucleation sites, which are dislocations (Figure 33f). Dislocation networks were also observed (Figure 33b). The presence of precipitates results in additional diffraction reflections around the reflections coming from the austenitic matrix.

**Figure 34:**
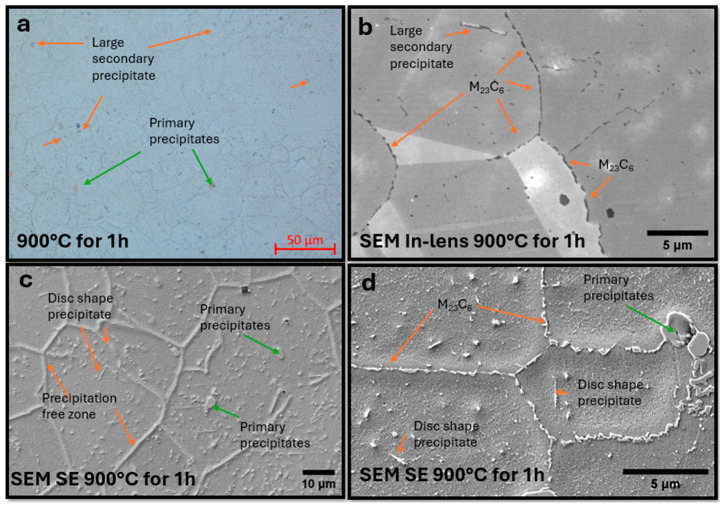
Microstructure of Sanicro 25 annealed at 900 °C for 1 h. Visible primary precipitates and relatively large secondary precipitates. LM (**a**,**b**), visible secondary precipitates inside the grains, a zone free of precipitates, and a significant amount of precipitates inside the grains. SEM (**c**,**d**).

The highest temperature at which the samples were annealed was 900 °C. After such treatment, there are significantly more precipitates in the microstructure (Figure 34), particularly within the grains. Carbide precipitates of the M_23_C_6_ type are still peeled off at grain boundaries, near which there are areas free of precipitates. Inside the grains, there are fine precipitates and larger precipitates with lamellar morphology.

**Figure 35:**
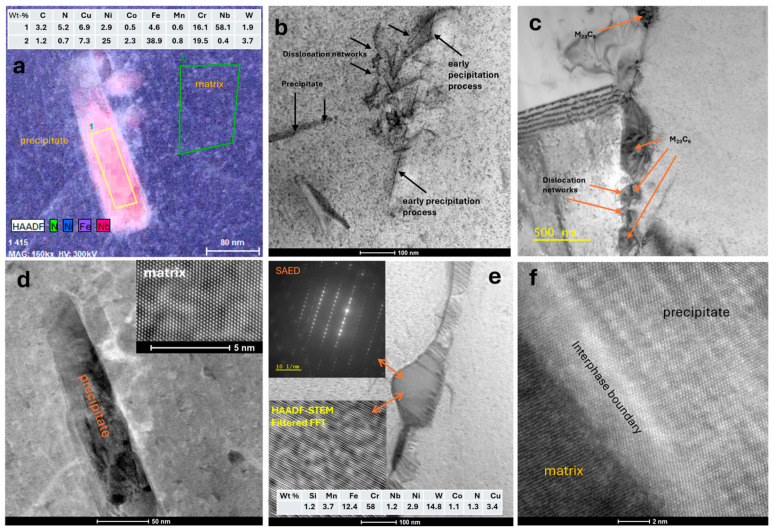
Microstructure of Sanicro 25 annealed for 1 h at 900 °C. Observed by TEM, complex precipitation and results of chemical composition microanalysis (STEM-EDS) (**a**). Precipitation in the initial stages and dislocation patterns (**b**), M_23_C_6_ carbides around which complex dislocation patterns form (**c**), complex precipitation, SAED, filtered STEM-HAADF, and phase boundary between the matrix and precipitation (**d**–**f**).

Studies by TEM confirmed the presence of complex carbonitrides (Figure 35a), whose chemical composition is shown in the table in Figure 35a. These lamellar precipitates were observed in the initial stages of the precipitation process. These precipitates had the same direction within a single grain, which suggests that the precipitate planes have a crystallographic relationship with the matrix planes. Dislocation systems were observed around the precipitates (Figure 35b). Dislocation networks (Figure 35c) were also observed. The observed precipitates exhibited an ordered crystal structure, and their crystal planes demonstrated partial coherence with the matrix.

At low temperatures, depletion of areas near grain boundaries in Cr was observed due to the presence of M_23_C_6_ carbides. In contrast, at higher temperatures, for which the rate of Cr diffusion is higher, the effect of grain boundary depletion of a significant extent, about 500 nm, was not observed. Probably longer annealing times of hundreds or thousands of hours would minimize this effect by allowing the diffusion of Cr from within the grains to areas near the boundaries and the consumption of carbon already present in the early stages of M_23_C_6_ carbide formation. Coherent lamellar precipitates were observed in the microstructure, suggesting the presence of crystalline relationships between the matrix and precipitates. However, Cu-rich precipitates, which were observed in samples that had been oxidized for an extended period, were not observed after annealing at temperatures up to 900 °C. Previous studies indicated that these Cu-rich precipitates are coherent with the matrix and, even under long times (20,000 h), have relatively small sizes in the order of tens of nm [8,9,16]. Cu-rich precipitates are coherent with the matrix due to the small differences in the elemental cell parameters of the austenitic matrix and Cu. Thus, it is possible that in the initial stages of precipitate formation, the precipitates have small sizes that do not allow their experimental confirmation. Precipitates of this kind are also not predicted by the thermodynamic simulations performed.

**Figure 36:**
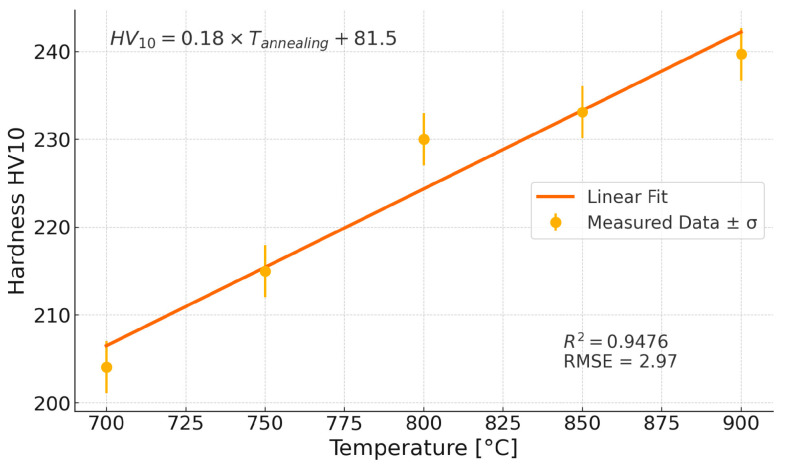
Mean hardness of Sanicro 25 as a function of annealing temperature.

Tests of HV10 hardness were carried out and showed an increase in hardness as the annealing temperature increased. The results of the hardness measurements of the samples are shown in Figure 36. The hardness increases by approximately 17% from 205 HV10 for the sample in its delivery condition to 240 HV10 for the sample annealed for one hour at 900 °C due to the precipitation process of strengthening phases from a supersaturated solid solution. A linear function can describe the increase in hardness when annealed for one hour:

$$HV10=0.18\times T_{annealing}+81.5,\;R^{2}=0.94;\;\mathrm{RMSE}=2.97$$

## 4. Conclusions

- Simulation of crystallization using the Scheil method indicates that full crystallization of Sanicro 25 steel will occur at 1209 °C. This is much lower than the temperature predicted by equilibrium calculations and lower than the supersaturation temperature during standard heat treatment of this steel, which is 1210 °C.
- It was possible to determine the kinetics of phase transformations as a function of time at elevated temperature (time equivalent, related to 900 °C), assuming that precipitation and phase transformation processes are controlled by the diffusion of substitutional elements. Precipitation processes occur preferentially at dislocations and scar boundaries, and raising the temperature from 750 °C to 900 °C strongly intensifies the precipitation processes and the formation of larger secondary phases.
- During cooling from the supersaturation temperature in air to a temperature at which diffusion processes are negligible (200 is about 1200 s, compared to the cooling time in water of about 54 s). Cooling in the air could preoccupy phase precipitation processes after the supersaturation process.
- Prisma simulations indicate that even short release times lead to quite intense Sigma phase release.
- Already after one hour of annealing, also at 700 °C, precipitates of M_23_C_6_ carbides were observed at grain boundaries. In the vicinity of the precipitates, a depletion of Cr in the matrix was observed to a depth of approximately 500 nm. Observation of the carbides due to their size is not directly possible using SEM techniques. Precipitates of this size, located at the grain boundaries, will be difficult to observe by TEM and microscopy techniques in general, due to the strong stress and diffraction contrast changes that occur in these areas as a consequence of the different orientations of adjacent grains.
- The simulations show significant depletion of the matrix in Cr with the presence of Cr-rich precipitates of small size. Analysis of the chemical composition using SEM-EDS and TEM-EDS techniques does not confirm a significant decrease in Cr content in the matrix. Thus, the precipitates may be so small that they are located in the volume that generates the characteristic radiation spectrum, so that the result obtained comes from both the matrix and precipitates.
- Pisma’s simulations correlate quite well with experimental observations on the kinetics of transformations. However, it should be emphasized that, in general, the phase sizes predicted by the simulations are smaller than the precipitates observed experimentally. In addition, during calculations, due to the large number of alloying elements, some simulations break down, especially when trying to simulate full cooling after supersaturation to room temperature.
- Thermodynamic simulations and experimental results demonstrated that increasing the annealing temperature from 750 to 900 °C significantly intensified precipitation processes in Sanicro 25 steel. Specifically, the simulations showed that the volume fraction of the Sigma phase decreased from approximately 17.5% at 700 to around 3.5% at 900 °C, while experimentally observed carbide (M_23_C_6_) reached sizes significantly larger than those predicted by the simulations (30 nm simulated vs. experimentally observed above 100 nm at higher temperatures). This indicates that higher annealing temperatures accelerate precipitation kinetics and result in precipitates considerably larger than computationally predicted.

## Figures and Tables

**Table 1:** Chemical composition of Sanicro 25 (wt%), manufacturer test certificate.

Ni	Cr	W	Cu	Co	Mn	Nb	N	Si	C	P + S	B	Fe
25.35	22.35	3.37	2.98	1.44	0.51	0.49	0.23	0.18	0.064	<0.016	0.003	Bal.

**Table 2:** Values of pre-exponential factor D_0_ and diffusion activation energy Q for selected elements in ferritic and austenitic matrix [32,33,34].

Element	Matrix	Pre-Exponential Factor D_0_ [m^2^/s]	Activation Energy of Diffusion Q [kJ/mol]
Cu	Austenitic	0.0001	280
Cu	Ferritic	0.00005	250
Ni	Austenitic	0.00062	300
Ni	Ferritic	0.0004	270
Cr	Austenitic	0.0025	310
Cr	Ferritic	0.002	300
W	Austenitic	0.001	320
C	Ferritic	0.00002	80
C	Austenitic	0.00002	142

**Table 3:** Chemical composition of Sanicro 25 after crystallization, determined by Scheil’s method.

		Chemical Composition wt%									
Volume Fraction, %	Phase	Fe	Mn	Cr	Ni	W	Co	Nb	Cu	C	N
97.10	Matrix (austenite)	39.4	0.9	27.3	22.6	5.7	1.2	0.1	2.5	0.1	0.3
1.10	(Cr. Nb)N	4.8	0.1	68.0	0.5	10.3	0.0	7.1		1.5	7.7
0.03	M_23_ C_6_	12.1	0.3	55.7	1.9	25.3	0.1			4.6	
0.30	Sigma	28.1	0.4	30.0	9.4	31.4	0.8	0.1			

**Table 4:** Equilibrium phase composition at 1210 °C, result of single-point Thermo-Calc calculations.

		Chemical Composition wt%									
Volume Fraction, %	Phase	Fe	Mn	Cr	Ni	W	Co	Nb	Cu	C	N
99.32	Matrix (austenite)	43.4	0.005	24.4	24.6	1	1.2	0.00052	2.6	0.01	0.7
0.06	FCC (Cr. Nb)N	-	-	13	-	-	-	36	-	15	33

## Data Availability

The data presented in this study are available on request from the corresponding author due to large amount of data.
